# Melanin protects *Cryptococcus neoformans* from spaceflight effects

**DOI:** 10.1111/1758-2229.13078

**Published:** 2022-07-19

**Authors:** Radames J. B. Cordero, Quigly Dragotakes, Phyllis J. Friello, Arturo Casadevall

**Affiliations:** ^1^ Molecular Microbiology and Immunology Department Johns Hopkins Bloomberg School of Public Health Baltimore MD 21205 USA; ^2^ Space Center Houston TX 77058 USA

## Abstract

As human activity in space continues to increase, understanding how biological assets respond to spaceflight conditions is becoming more important. Spaceflight conditions include exposure to ionizing radiation, microgravity, spacecraft vibrations and hypervelocity; all of which can affect the viability of biological organisms. Previous studies have shown that melanin‐producing fungi are capable of surviving the vacuum of space and Mars‐simulated conditions in Low Earth Orbit. This survival has been associated in part with the protective effects of melanin, but a comparison of fungal viability in the presence or absence of melanin following spaceflight has never been tested. In this study, we evaluated the protective effects of melanin by comparing the viability of melanized and non‐melanized clones of *Cryptococcus neoformans* yeasts following a roundtrip to the International Space Station. Yeast colonies were placed inside two MixStix silicone tubes; one stayed on Earth and the other was transported inside for 29 days before returning to Earth. Post‐flight analysis based on colony‐forming unit numbers shows that melanized yeast viability was 50% higher than non‐melanized yeasts, while no difference was observed between the Earth‐bound control samples. The results suggest that fungal melanin could increase the lifespan of biological assets in space.

## Introduction

Ionizing radiation microgravity, spacecraft vibrations, hypervelocity and the potential synergistic interactions between these environmental factors can harm biological cargo during spaceflight (Horneck *et al*., 2010; Huang *et al*., 2018). For example, ionizing radiation can damage cells by ionizing intracellular biomolecules, changing their structure and function, which can result in cellular and tissue damage. Organisms on Earth are shielded from much of this ionizing radiation by the Earth's protective atmosphere and magnetic field which deflects, and traps charged particles in the Van Allen belts. The International Space Station orbits Earth at an average height of 400 km above the Earth, yet still, within the Van Allen belts, it is exposed to Solar Energetic Particles, Galactic Cosmic Rays, and trapped protons in the South Atlantic Anomaly (SAA). The absorbed doses for crews living on the International Space Station for 6–12‐month missions range from ~30 to 120 mGy (Patel *et al*., 2020). Beyond Earth's protective magnetosphere, astronauts travelling to the moon or Mars will be exposed to ionizing radiation for longer periods, which may lead to serious health problems (Delp *et al*., 2016; Elgart *et al*., 2018; Patel *et al*., 2020). As interest in spaceflight continues to grow, understanding the biological consequences of spaceflight factors is important to identify ways to protect biological assets, especially during long‐term space missions.

Microorganisms are useful models to study the biological effects of spaceflight and are ubiquitous in space habitats. Knowing how microorganisms respond to spaceflight conditions is also important because microbial adaptations could potentially lead to increased risks to astronaut health (Wilson *et al*., 2007; Rosenzweig *et al*., 2010; Crabbé *et al*., 2013), and because of their value in space biomanufacturing (Lam *et al*., 1998, 2002; Benoit *et al*., 2006; Ball *et al*., 2021; Bijlani *et al*., 2021). Multiple spaceflight experiments onboard the International Space Station with bacteria, fungi and archaea species suggest that spaceflight can affect microbial growth and metabolism (Horneck *et al*., 2010; Huang *et al*., 2018; Milojevic and Weckwerth, 2020; Bijlani *et al*., 2021). Other experiments have even looked at the capacity of microorganisms to survive outside the International Space Station. Fungal species of *Aspergillus*, *Penicillium* and *Cryomyces* have been shown to survive long periods of exposure to the harsh vacuum, high‐energy UV rays and extreme temperature fluctuations in Low Earth Orbit (Horneck *et al*., 1999; Panitz *et al*., 2001; Onofri *et al*., 2012, 2019; Pacelli *et al*., 2019). One common characteristic of these fungal species is that they all produce the pigment melanin.

Melanins are a special class of multifunctional polymers responsible for the dark colouration in animals, plants, fungi and bacteria (Cordero and Casadevall, 2020). These biopolymers are considered natural sunscreens based on their ability to absorb the ultraviolet sun rays that penetrate Earth's atmosphere and damage the human skin (Brenner and Hearing, 2008). In fungi, melanins contribute to host infection and protect from multiple environmental stressors (Cordero and Casadevall, 2017; Smith and Casadevall, 2019). Studies comparing the survival of melanized and non‐melanized fungal species show that the presence of melanin is associated with increased survival following exposure to different types of physical and chemical stress factors, including ionizing radiation including X‐rays, γ‐rays and particulate radiation (Robertson *et al*., 2012; Shuryak *et al*., 2015; Pacelli *et al*., 2017; Cortesão *et al*., 2020); ultraviolet radiation (Wang and Casadevall, 1994a); oxidative stress (Jacobson and Tinnell, 1993; Wang and Casadevall, 1994b; Jahn *et al*., 2000); heat and cold stress (Rehnstrom and Free, 1996; Rosas and Casadevall, 1997; Paolo *et al*., 2006); metal toxicity (García‐Rivera and Casadevall, 2001); osmotic stress (Kogej *et al*., 2007; Fernandez and Koide, 2013; Kejžar *et al*., 2013). Based on these studies we hypothesized that the yeast specimens containing melanin would be protected from spaceflight effects during transit and residence on the International Space Station and will produce more living colonies than non‐melanized yeast specimens. To test the hypothesis, we use the encapsulated fungus *Cryptococcus neoformans*, a light‐coloured environmental basidiomycete that can turn black by coating its cell wall with melanin nanoparticles only if grown in the presence of a melanin precursor such as l‐3,4‐dihydroxyphenylalanine (l‐DOPA) (Garcia‐Rivera *et al*., 2005; Camacho *et al*., 2019). Melanized and non‐melanized *C*. *neoformans* clones were grown in a solid medium and packed inside two MixStix tubes; one was sent to the International Space Station for 29 days aboard the spacecraft while the other remained on Earth as control. The number of living yeast colonies on both specimens was counted and compared upon returning to Earth. The hypothesis would be rejected in any case in which the number of living colonies on the non‐melanized sample is not less than the number on the melanized specimen.

## Results and discussion

**Fig. 1:**
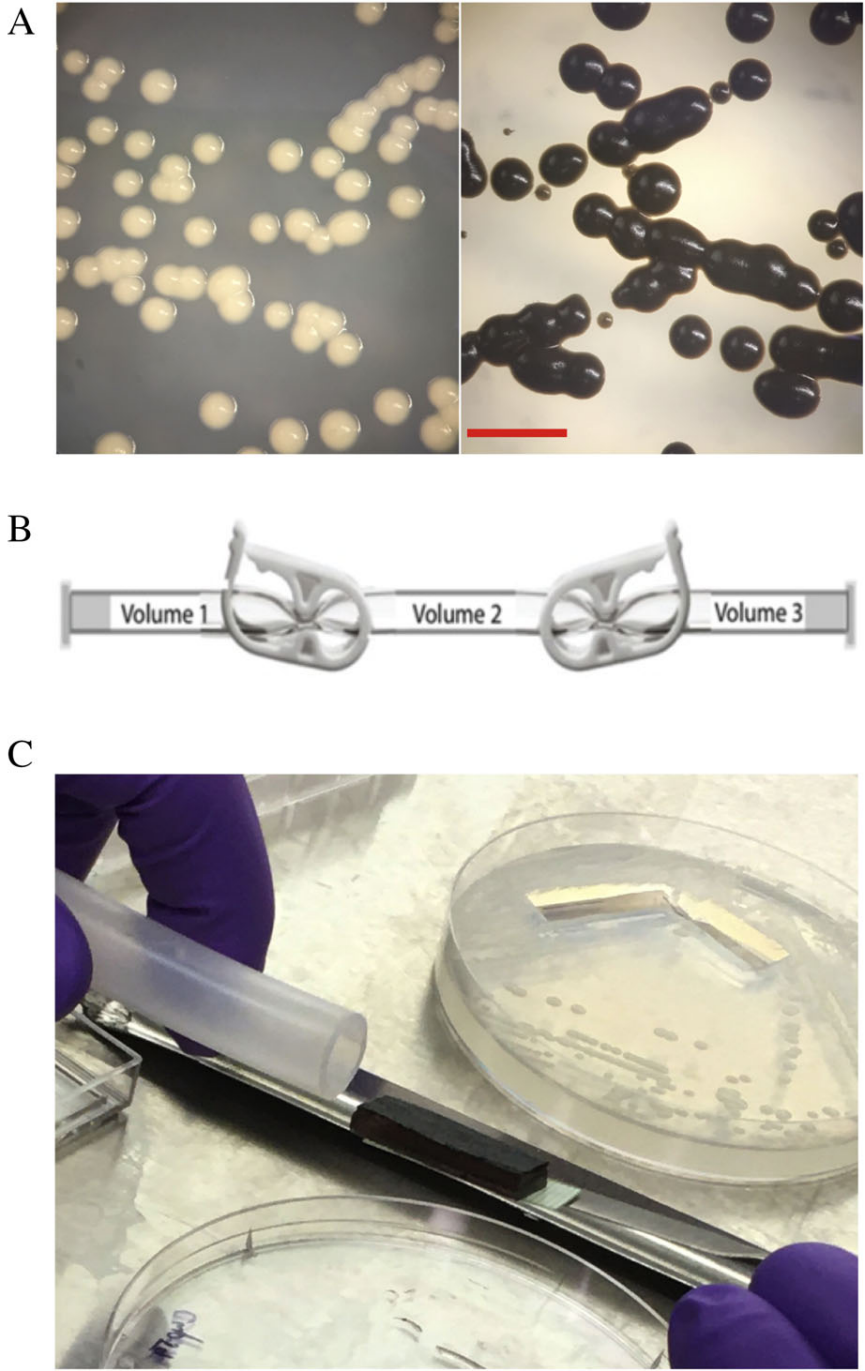
Preparation of flight samples. A. Examples of non‐melanized and melanized clones of *C*. *neoformans* grown in solid minimal media. Scale bar, 1 cm. B. The MixStix is a 10 ml parylene‐coated silicon tube used to carry the fungal samples. It was compartmentalized into three volumes using two Clamps (A and B). Volume 1 contained two rectangular pieces of agar containing living melanized and non‐melanized *C*. *neoformans* lawns (approximately ~10^8^ cells). Volume 2 and 3 contained an uncoated and melanin‐coated dosimeter (RADTriage50 radiation sensor card) respectively. The sensor card was cut to fit the diameter of the MixStix tube. However, no useful data could be obtained from the sensor cards once cut. C. Shows a rectangular piece of melanized fungal lawn being placed inside the MixStix‐Volume 1.

**Fig. 2:**
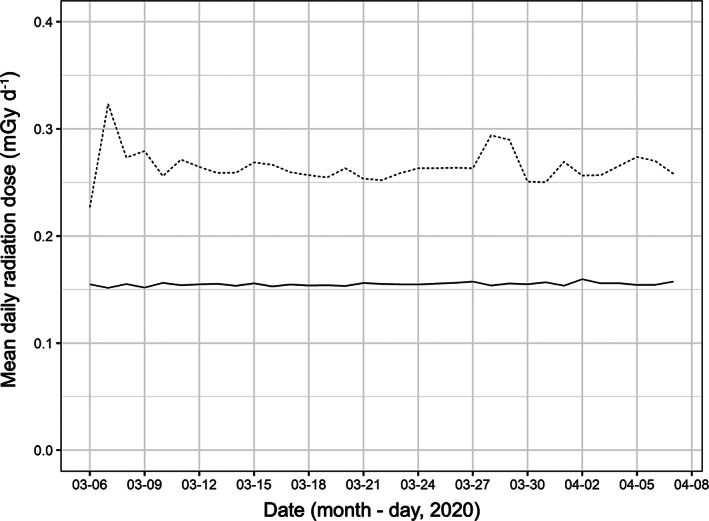
Mean daily radiation dose detected at the Japanese Pressurized Module by the International Space Station Radiation Environment Monitor 2 (REM2). The dashed line corresponds to the South Atlantic Anomaly (SAA) and the solid line is Galactic Cosmic Rays (GCR) in milli Gray per day. Data were provided by the Space Radiation Analysis Group (SRAG).

Melanized and non‐melanized clones of *Cryptococcus neoformans* strain H99 were grown as lawns on solid minimal media comprised of 20% (vol./wt.) agar, 10 mM MgSO_4_, 29.3 mM KH_2_PO_4_, 13 mM glycine, 3 μM thiamine‐HCl, adjusted to pH 5.5, with or without 1 mM l‐DOPA respectively. Fungal lawns were produced by spreading 50 μl of a 48‐h pre‐culture in Sabouraud‐dextrose broth on the minimal media agar plate using a sterile loop and allowing it to grow for 48 h inside an incubator set at 30°C. On February 12, 2020, rectangular pieces of the lawn‐agar were cut with sterile scalpels and lifted into the MixStix (Nanoracks); a 10 ml parylene‐coated silicon tube, divided into three compartments using two clamps (Fig. 1). Volume 1 contained the melanized and non‐melanized *C*. *neoformans* lawns grown on agar. Volume 2 and 3 contained an uncoated and melanin‐coated dosimeter (RADTriage50 radiation sensor card) respectively. However, no useful data could be obtained from either sensor card. Two identical MixStix were prepared; one for deployment to the International Space Station and one as an Earth control stored on the laboratory bench at 24°C and 54% relative humidity. The ISS‐bound MixStix was placed inside a Styrofoam box aboard the SpaceX CRS‐20 payload fairing structure made of a 2.5 cm thick aluminium honeycomb core surrounded by carbon fibre sheet piles; all of which provided insulation for the samples during flight. The samples launched on March 6, 2020, and arrived at the International Space Station on March 9, 2020. The MixStix was stored on the Japanese Experimental Module (KIBO) maintained at a temperature of 18.3°C–26.7°C, a pressure of 1 atm, and relative humidity of 25%–70% (Japan Aerospace Exploration Agency, 2007). The mean daily radiation dose detected at the Japanese Pressurized Module was 154.9 ± 1.6 μGy d^−1^ for Galactic Cosmic Radiation and 264.7 ± 15.9 mGy d^−1^ for SAA (Fig. 2), consistent with a previous report (Berger *et al*., 2012). Added together, the total radiation dose exposure was approximately 14 mGy. The MixStix departed the International Space Station on the cargo vehicle and returned to Earth on April 9, 2020. The samples arrived at the Johns Hopkins Bloomberg School of Public Health for viability analysis on April 17, 2020.

**Fig. 3:**
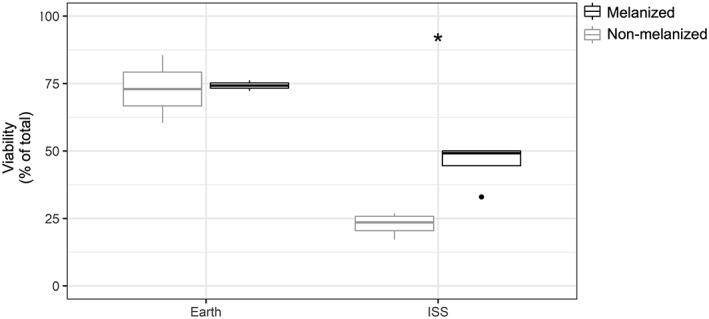
Boxplots showing viability differences between melanized and non‐melanized yeasts following roundtrip to the International Space Station (ISS) and Earth‐bound control. Cell viability was evaluated by enumerating the CFUs of Earth‐bound and ISS‐flown yeast samples relative to the total number of cells inoculated; 500 and 250 cells per plate in quadruplicates (eight plates per sample condition). The viability of samples was analysed four independent times, but we had contamination issues with the Earth‐bound sample on two occasions. Hence, the percent viability of the ISS‐bound results corresponds to four biological replicas and the Earth‐bound results correspond to two replicas. The bars on the boxplots are 95% confident intervals. The black dot represents data values outside the confidence interval. Asterisks denote *P* < 0.05 comparing melanized and non‐melanized samples via ANOVA with Tukey tests.

Immediately upon return, the lawn‐agar pieces were removed from the MixStix and placed inside sterile 50‐ml tubes. Five millilitres of fresh Sabouraud‐dextrose broth was added to each tube to gently detach and re‐suspend the yeast cells from the agar using a P1000 micropipette. The total number of cells per sample was approximately 1.7 × 10^8^ for the non‐melanized ISS‐bound, 1.2 × 10^8^ for the melanized ISS‐bound, 5.2 × 10^8^ for the non‐melanized Earth‐bound and 6.8 × 10^8^ for the melanized Earth‐bound. For viability analysis, the cell suspensions were enumerated using a haemocytometer to calculate the number of cells per volume and perform serial dilutions to inoculate 500 and 250 cells on Sabouraud agar plates in quadruplicates. CFUs were enumerated after 2 days of incubation at 30°C. Percent viability was calculated as CFUs relative to the total number of cells inoculated. CFU analysis showed that yeasts exposed to the International Space Station interior environment exhibited lower viability than the Earth‐bound control group (Fig. 3). While Earth‐bound melanized and non‐melanized yeasts exhibited similar viability rates, melanized cells transported to the International Space Station showed ~50% higher viability than non‐melanized clones. We do not know how melanin specifically contributed to the higher viability of the melanized sample but we note that the yeasts were exposed to a variety of insults such as ionizing radiation, microgravity, hypervelocity, and so on. The observed difference in viability suggests that melanin is associated with higher survival in *Cryptococcus* yeasts following exposure to the International Space Station environment as well as the impact of the launch and return trips.

**Fig. 4:**
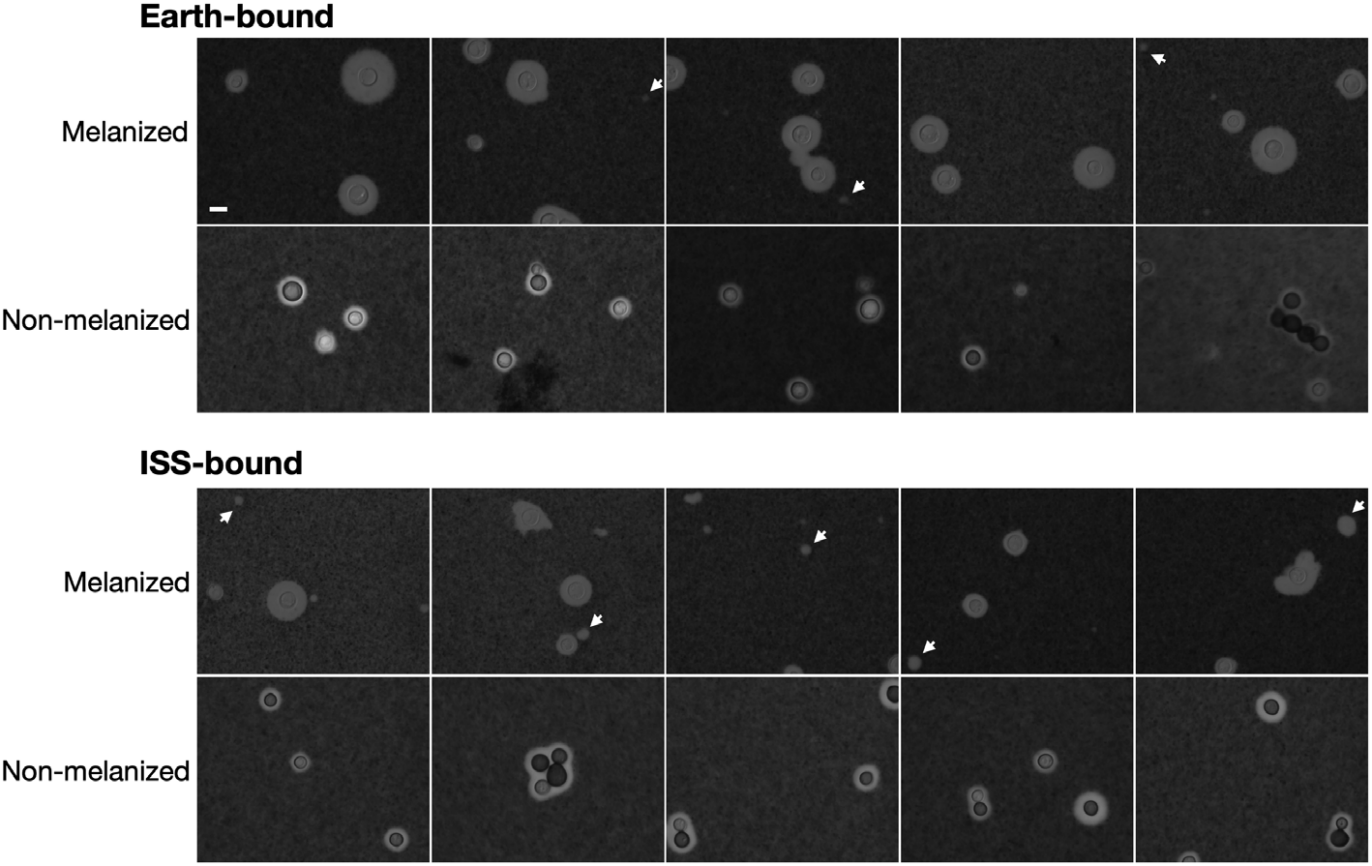
Light microscopy images of melanized and non‐melanized yeast cells following spaceflight and the Earth‐bound control sample. Light microscopy of Earth and International Space Station (ISS) *C*. *neoformans* specimens. Objective: 100× oil immersion. Scale bar, 5 μm. All images were captured using the same exposure time. Differences in the background darkness behind cells are not due to differences in exposure time but to the inherent heterogeneity of the counterstain (India Ink) on mounted glass slides. White arrows show examples of capsule fragments that were more prevalent in the non‐melanized sample, especially in the ISS‐bound.

Post‐flight microscopy analysis showed *C*. *neoformans* with normal morphology as well as yeasts devoid of, or with damaged, polysaccharide capsules (Fig. 4). We also observed cell‐dissociated fragments of capsules under India‐ink counterstaining. These capsular deformations were almost exclusively observed in the non‐melanized samples and much more frequently in the ISS‐bound sample. By visual inspection, we counted 40 capsule fragments for every 30 cells in the ISS‐bound sample versus 21 fragments per 47 cells in the Earth‐bound sample. One potential cause for these capsular deformations may be related to polysaccharide ablations caused by ionizing radiation exposure (Bryan *et al*., 2005). Ablation of *Cryptococcus* capsular polysaccharides can be observed following exposure to 3 Gy of radiation (Bryan *et al*., 2005), which is much higher than the ~14 mGy total radiation detected during the 29 days aboard the Japanese Experimental Module. We also note that the actual radiation experience by the yeast cells may have been lower considering the radiation shielding properties of silicon polymers (Nagaraja *et al*., 2020). Although no previous studies have studied the impact of equivalent low radiation doses, it may be possible that even such low radiation doses could cause polysaccharide ablations considering these are natural radiation energies exposed for 29 consecutive days in an actual space environment with the combination of other interacting physicochemical stressors (i.e. microgravity, hypervelocity). Ground irradiation experiments using human‐engineered radiation sources with equivalent radiation exposures (~155–265 μGy d^−1^) are needed to confirm that the capsule deformations observed here are in fact due to spaceflight‐related radiation effects. Other forces like microgravity and/or hypervelocity may also contribute to capsule damage; however, the effects of microgravity or high g‐force on capsule morphology are yet to be explored. The lack of capsule deformation events in the melanized samples may suggest that melanin, directly or indirectly, renders cells less susceptible to capsule deformation during spaceflight exposure.

**Fig. 5:**
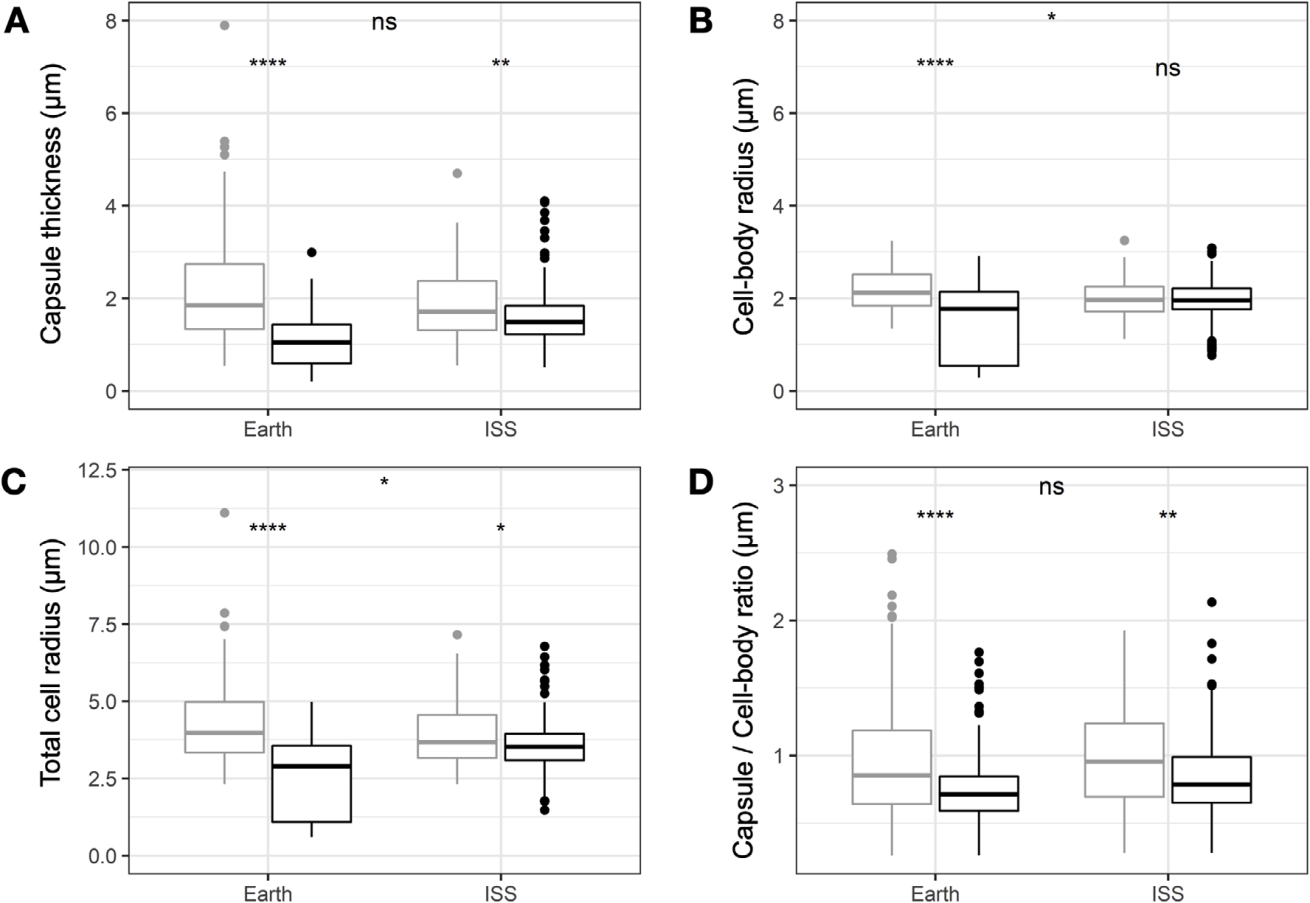
Boxplots showing cellular dimensions measured from light microscopy images. A. Capsule radius (μm). B. Cell body radius (μm). C. Total cell radius (μm). D. The capsule to cell body ratio. Measurements were done manually using the ImageJ software as previously described (Cordero *et al*., 2011). The bars on the boxplots are 95% confident intervals. Dots represent data values outside the confidence interval. Middle asterisks denote comparisons between International Space Station (ISS) and Earth‐bound using ANOVA with Tukey tests, while left and right asterisks denote comparisons between melanized and non‐melanized samples (*n* = 120 cells per group; one biological replicate). NS = not significant; and *, ** and *** denote *P* < 0.05, *P* < 0.01 and *P* < 0.001 respectively.

On average, non‐melanized cells showed larger capsules than melanized cells (Fig. 5A), consistent with previous observations (Vij *et al*., 2018). Melanized cells exposed to spaceflight conditions showed larger capsules sizes than the Earth‐bound control cells (*P* = 0.0000001). The relative increase in capsule size observed in melanized cells exposed to the International Space Station is interesting since the opposite was observed between non‐melanized cells. While the average cell body radius of non‐melanized cells was larger than melanized cells in the Earth‐bound control, this size difference was lost in the ISS‐bound samples (Fig. 5B). The average total cell radius (Fig. 5C) and capsule to cell body ratio (Fig. 5D) follow a similar trend to capsule sizes. Although we cannot explain the mechanism underlying these morphological changes, the data suggest that spaceflight exposure is associated with changes in capsule and cell body dimensions *Cryptococcus neoformans*.

In conclusion, the results suggest that melanin is associated with higher viability of *C*. *neoformans* yeast cells following spaceflight exposure. We note that our results are biologically plausible based on what is known about the protective properties of melanin. It is important to note that, in addition to melanin‐mediated protection effects from spaceflight conditions, it is also possible that the higher viability in melanized cells could also be related to the energy‐harvesting properties of fungal melanin following exposure to ionizing radiation (Dadachova *et al*., 2007). Since spaceflight exposure involves a combination of stressors, future ground experiments that can separate some of these effects are of interest; for example, measuring the viability of melanized versus non‐melanized cells following exposure to comparable low radiation doses, simulated microgravity, and high g‐force, individually. Future space flight experiments should consider other melanized and non‐melanized species, embedded radiation‐temperature‐humidity sensors, and other types of viability assessments linked to metabolic and genetic changes to better understand the protective effects of melanin during spaceflight.

## Conflict of Interest

R.J.B.C., A.C. and Q.D. have financial interests in MelaTech, LLC, a biotech startup dedicated to the large‐scale production of fungal melanin.
